# Function of the R2R3-MYB Transcription Factors in *Dalbergia odorifera* and Their Relationship with Heartwood Formation

**DOI:** 10.3390/ijms241512430

**Published:** 2023-08-04

**Authors:** Ruoke Ma, Jia Luo, Weijie Wang, Tianqi Song, Yunlin Fu

**Affiliations:** 1 Key Laboratory of National Forestry and Grassland Administration for Fast-Growing Tree Breeding and Cultivation in Central and Southern China, College of Forestry, Guangxi University, Nanning 530004, China; 1909401001@st.gxu.edu.cn (R.M.); 1909392013@st.gxu.edu.cn (J.L.); 2009392029@st.gxu.edu.cn (W.W.); 2 College of Agronomy, Northwest A&F University, Xianyang 712000, China; songtq@nwafu.edu.cn

**Keywords:** R2R3-MYB, *Dalbergia odorifera*, heartwood formation, comparative phylogenetic analysis

## Abstract

R2R3-MYB transcription factors (TFs) form one of the most important TF families involved in regulating various physiological functions in plants. The heartwood of *Dalbergia odorifera* is a kind of high-grade mahogany and valuable herbal medicine with wide application. However, the role of R2R3-MYB genes in the growth and development of *D. odorifera*, especially their relevance to heartwood formation, has not been revealed. A total of 126 R2R3-MYBs were screened from the *D. odorifera* genome and named DodMYB1-126 based on their location on 10 chromosomes. The collinearity results showed that purification selection was the main driving force for the evolution of the R2R3-MYB TFs family, and whole genome/fragment replication event was the main form for expanding the R2R3-MYB family, generating a divergence of gene structure and function. Comparative phylogenetic analysis classified the R2R3-MYB TFs into 33 subfamilies. S3-7,10,12-13,21 and N4-7 were extensively involved in the metabolic process; S9,13,16-19,24-25 and N1-3,8 were associated with the growth and development of *D. odorifera*. Based on the differential transcriptional expression levels of R2R3-MYBs in different tissues, DodMYB32, DodMYB55, and DodMYB89 were tentatively screened for involvement in the regulatory process of heartwood. Further studies have shown that the DodMYB89, localized in the nucleus, has transcriptional activation activity and is involved in regulating the biosynthesis of the secondary metabolites of heartwood by activating the promoters of the structural genes *DodI2’H* and *DodCOMT*. This study aimed to comprehensively analyze the functions of the R2R3-MYB TFs and screen for candidate genes that might be involved in heartwood formation of *D. odorifera*.

## 1. Introduction

*Dalbergia odorifera* T. Chen (*D. odorifera*), commonly known as Hainan Huanghuali, is a semi-deciduous tree belonging to the Fabaceae family. It is native to Hainan, China and has been introduced to the Fujian, Guangdong, Yunnan, and Zhejiang regions of China, with a large artificial plantation area that follows an increasing trend every year [1]. The heartwood of the xylem of *D. odorifera* has a wide range of application. The heartwood is hard and resistant to decay, has a beautiful pattern and color, and has a pleasant aroma. It is considered the best wood for high-grade furniture because of all these characteristics. Heartwood is rich in a large number of active medicinal ingredients. It is a valuable Chinese medicine called “Jiangxiang”, which can be used for treating cardiovascular and rheumatic diseases [2]. Regulating heartwood formation is the key to increasing the utilization value of *D. odorifera*. Because of its excellent quality and important application value, *D. odorifera* is considered an ideal model species for studying heartwood, especially the precious mahogany heartwood. The publication of *D. odorifera* genomic data has prompted researchers to explore the molecular mechanisms regulating the formation of xylem heartwood [3].

TFs regulate the programmed expression of target genes by specifically binding to cis-acting elements in the promoter region [4]. TFs can be classified into different types of gene families depending on the sequence of the DNA-binding structural domain contained in their protein sequences. The MYB transcription factor (v-MYB avian myeloid virus oncogene homolog) family is one of the most important TFs in plants [5]. The most prominent feature of the MYB transcription factor family is the tandem association of 1–4 incompletely repeated DNA-binding structural domains (MYB structural domains) at the N-terminal region of the protein sequence. Each MYB structural domain repeat region (R) contains approximately 52 amino acid residues and forms a helix-turned-helical structure that specifically recognizes the DNA sequence of the target gene [6]. According to the number of repeats, MYB TFs are classified into four types: 1R-MYB (MYB-related), 2R-MYB (R2R3-MYB), 3R-MYB (R1R2R3-MYB), and 4R-MYB. Among them, 4R-MYB is the least abundant type and 3R-MYB type is the most abundant in animals [6]. Compared with the other three types, the R2R3-MYB transcription factor is the most abundant and widely studied type of MYB transcription factor in higher plants [7]. Many R2R3-MYB TFs have been identified due to the sequencing of a large number of plant genomes, such as the model plant *Arabidopsis thaliana* [8], the woody plants *Eucalyptus grandis* [9] and *Populus trichocarpa* [10], the gymnosperm *Picea abies*, and the basal angiosperm *Amborella. richopoda* [11]. More and more functions of R2R3-MYB genes are being gradually revealed, and according to the studies published in recent years, they can be divided into three main types of functions: (i) Regulation of cell differentiation and organ development, such as the differentiation of trichome formation and the development of organs such as seeds and flowers [12,13]; (ii) Regulation of secondary metabolic processes, such as the synthesis of flavonoids, anthocyanins, and stilbene [14,15]; (iii) Mediating signals in response to biotic and abiotic stresses, such as drought and salinity stresses [16]. Hence, R2R3-MYB TFs are indispensable key factors involved in life activities such as plant development and metabolism; however, the sequence characteristics and functions of the R2R3-MYB TFs in *D. odorifera* are unknown.

Based on the bioinformatics software applications, the physicochemical properties, conserved structural domains, phylogenetic levels, and expression patterns of the R2R3-MYB TF family in the *D. odorifera* genome were analyzed to investigate the following: (1) the physicochemical properties, conserved structural domains, and other genetic characteristics of *D. odorifera* R2R3-MYB proteins; (2) the phylogenetic and functional predictions of *D. odorifera* R2R3-MYB proteins; and (3) the specific expression levels of R2R3-MYB genes in different tissues in the *D. odorifera* genome. This study aimed to further analyze the structural characteristics of the *D. odorifera* R2R3-MYB gene and its evolutionary relationship, to elucidate the function of MYB gene, to find the molecular mechanism regulating the formation of xylem heartwood, and to contribute to the development of *D. odorifera*.

## 2. Results

### 2.1. Sequence Characterization and Physicochemical Properties of D. odorifera R2R3-MYB Transcription Factors

**Figure 1:**
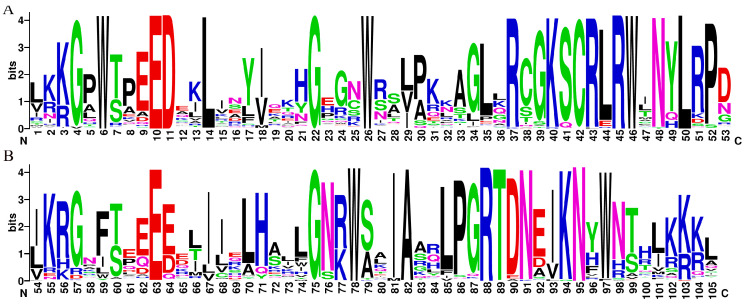
Seqlogo plots of the MYB structural domains of degenerate *D.odorifera* R2 (**A**) and R3 (**B**). The score of amino acids (Bits) indicates the frequency of its occurrence at this locus.

The MYB structural domain-containing sequences (comprising 4R, 3R, 1R, and 2R-MYB TFs) were screened from the *D. odorifera* genome using the Hidden Markov Model (PF00249) as a search file. The members of the R2R3-MYB gene family of *D. odorifera* were identified based on sequence features. These members were named DodMYB1-126 based on their position on the chromosome (Figure S1). Sequence logos were created for R2 and R3 of *D. odorifera* R2R3-MYB by manually intercepting the conserved structural domain information obtained with reference to the amino acid sequence information of the structural domain of *A. thaliana* R2R3-MYB after multiple-sequence alignment (Figure S2). As shown in Figure 1, the conserved motifs at the N-terminus of the R2R3-MYB protein were [W]-X3-[ED]-X2-L-X7-[G]-X3-[W]-X10-[R]-X2-[KSCRLRW]-X32-[W]-X2-[IA]-X5-[RTDN]-X2-KN-X-W. These conserved residue-distribution characteristics of the R2R3-MYB structural domain of *D. odorifera* were very similar to those of other species, indicating the conserved nature of the MYB gene during the evolution of the plant lineage. Among these (Figure 1), the R2 and R3 repeat regions had a triplet structure having three highly conserved tryptophan residues (W), a typical hallmark of plant MYB proteins, indicating the importance of conserved W residues in sequence-specific DNA binding and the maintenance of the helix-turn-helix structure. The R3 repeat has a similar conserved structure to the R2 repeat but is slightly different. The W residue at position 59 is mainly substituted by phenylalanine (F), followed by isoleucine (I) and leucine (L) in *D. odorifera* R2R3-MYB TFs (Figure 1). The last W residue at position 97 of the R3 repeat region is also not fully conserved. The W residues were substituted by F residues in a small number of sequences (DodMYB25, DodMYB51, and DodMYB124). This phenomenon of the MYB protein in *D. odorifera* is also present in other plants, such as soybeans and pears [17,18], which may be related to the specificity of the promoter sequence of the target gene, but the underlying mechanism needs to be further explored.

The physicochemical properties of the DodR2R3-MYB TFs in *D. odorifera* differ in amino acid number, isoelectric point, and relative molecular mass (Table S1). The 126 DodR2R3-MYB proteins had a minimum amino acid number of 136 (DodMYB58) and a maximum amino acid number of 565 (DodMYB60). The instability index ranged from 39.85 to 71.11, and only one protein (DodMYB67) belonged to the category of stable proteins (instability index less than 40), indicating that the *D. odorifera* R2R3-MYB transcription factor family as a whole was in an unstable state and the proteins were easily degraded (Table S1). The isoelectric points (*pI*) of the DodR2R3-MYB proteins ranged from 4.78 to 10.29, with 77 proteins having a *pI* of less than 7.0 and 49 proteins having a *pI* greater than 7.0, most of which were acidic proteins. Based on the predicted hydrophilicity of the proteins, the hydrophilic index of R2R3-MYB of *D. odorifera* was found to be less than 0, and it was assumed that these proteins were unstable hydrophilic proteins. The subcellular localization prediction revealed that only DodMYB6 was localized on chloroplasts, while the rest of the proteins were localized in the nucleus (Table S1).

### 2.2. Gene Structure and Motif Analysis of D. odorifera R2R3-MYB Transcription Factor Family

**Figure 2:**
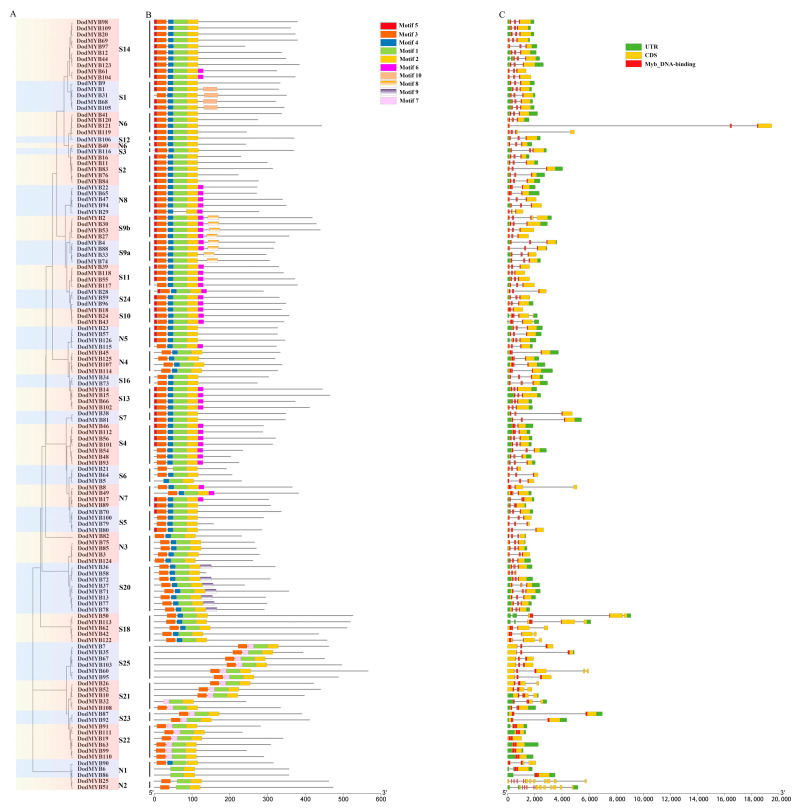
Motif sequence and gene structure of R2R3-MYB TFs in *D. odorifera.* (**A**). Phylogenetic tree built using 126 *D. odorifera* R2R3-MYB proteins. The tree contains 33 subfamilies marked with colored backgrounds. (**B**). Architectures of conserved protein motifs. The different colored boxes indicate distinct motifs and their corresponding positions in each R2R3-MYB protein sequence. The detailed characteristics of each motif are shown in Figure S3. (**C**). Exon/intron structures of *D. odorifera* R2R3-MYBs.

The diversity of exon/intron structures in the MYB gene of *D. odorifera* is a reflection of functional differentiation during the evolution of the R2R3-MYB family. As shown in Figure 2, except for S22 (DodMYB19, DodMYB63, DodMYB99, DodMYB110, and DodMYB111), which had no introns, the remaining 121 2R-MYB genes had different numbers of introns, ranging from 1 to 12 (the most common case is to contain 2), and the coding sequences were disrupted by introns. Moreover, the exon sizes of *D. odorifera* R2R3-MYBs also differed. In general, exons 1 and 2 encode almost the entire MYB domain, and exon 3 encodes the C-terminal region and the remaining domain of the R2R3-MYB protein (Figure 2). Exon 3 is more diverse in terms of length relative to exons 1 and 2. Similar findings regarding the exon length of *D. odorifera* R2R3-MYBs were reported in other plants such as *A. thaliana* [8], *P. trichocarpa* [10], and *E. grandis* [9].

A total of 10 conserved motifs, ranging in length from 8 to 36 amino acids, were identified in 126 *D. odorifera* R2R3-MYB TFs (Figure 2 and Figure S3). Three highly conserved motifs (motif1, motif2, and motif3) located at the N-terminus were important parts for the two MYB repeats and were present in almost all protein sequences. At the C-terminus of MYB proteins, most members of the same subfamily had the same number and type of motifs that might have similar functions within the subfamily, but there were large differences between subfamilies, implying the functional differentiation of R2R3-MYB genes [10]. For example, motif8 is only present in members of the S9 subfamily associated with tissue differentiation functions. In conclusion, although the motifs from different subfamilies differed in type and number, the characteristics of protein sequences of the same subfamily were conserved and consistent with the results of phylogenetic tree analysis (Figure 2), corroborating the subfamily classification results.

### 2.3. Chromosomal Localization and Synteny Analysis of D. odorifera R2R3-MYB TFs

**Figure 3:**
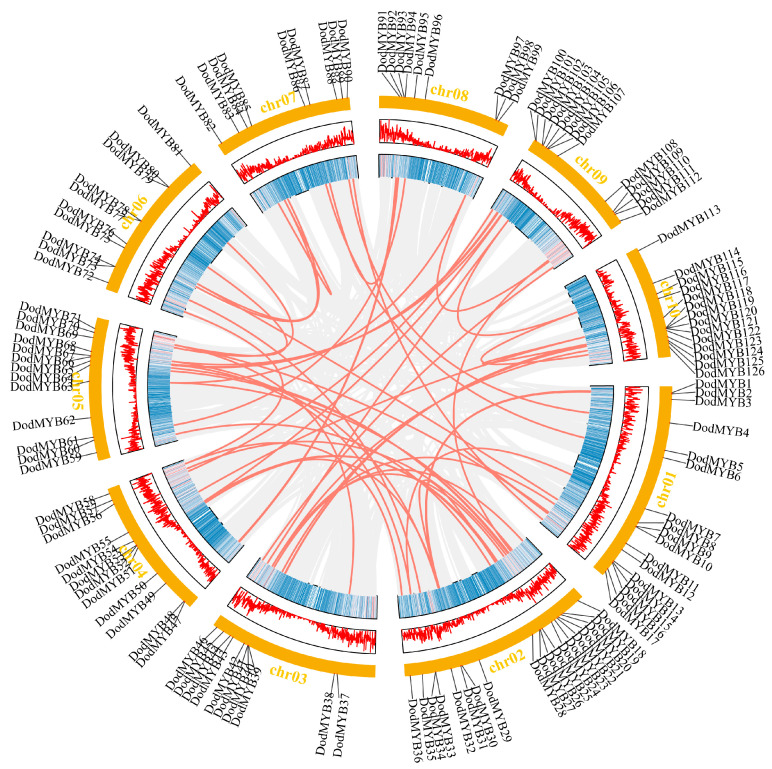
Schematic representations of collinear relationships and the location of *D. odorifera* R2R3-MYB TFs on chromosomes. Gray and red lines represent all synteny blocks and duplicated MYB gene pairs in the *D. odorifera* genome, respectively. The corresponding number of each chromosome is shown above the chromosome.

Information on the location distribution of the R2R3-MYB gene on the chromosome backbone was obtained from the *D. odorifera* genome annotation file (Figure S1 and Figure 3). Among them, 126 TFs were unevenly distributed on 10 chromosomes, with the highest numbers on Chr02 (19 members) and Chr01 (17 members) and the lowest number on chromosomes 7 and 8 (9 members). Secondly, even on the same chromosome, the distribution was uneven. Most R2R3-MYB genes were usually clustered on certain segments of chromosomes and showed a tendency to be distributed at both ends of the chromosomes, with less distribution in the central region of the chromosomes (Figure S1 and Figure 3). This type of nonuniform distribution pattern has also been found in other plants, such as *P. trichocarpa* [10] and *Physcomitrella patens* [19], and it is hypothesized that nonuniform replication events of *D. odorifera* chromosomal segments are the main cause of this distribution.

Gene duplication is an important driver of multigene family expansion and promotes the functional differentiation of gene families [20]. The *D. odorifera* genome underwent at least two rounds of whole-genome duplication [3], followed by fragment duplication, tandem duplication, or gene translocation events. The synteny analysis revealed 58 gene pairs with collinear relationships in the *D. odorifera* genome, involving 93 R2R3-MYB genes (Table S2). Among them, WGD or fragment duplication is the main form of expansion in the *D. odorifera* MYB gene family, distributed on 10 chromosomes, and plays a key role in the evolution of *D. odorifera*. Moreover, intra-chromosomal replication events in the *D. odorifera* genome were observed (Table S2). Two tandem duplication events were on chromosome 2 and one tandem duplication event was on chromosome 10. Non-synonymous and synonymous substitution ratios (Ka and Ks) were calculated for genes with collinear relationships to detect the drivers of the evolution of the *D. odorifera* R2R3-MYB gene family (Table S2). The results showed that the ratio of synonymous mutations was greater than the ratio of nonsynonymous mutations (Ka/Ks < 1) for almost all genes with collinearity relationships, indicating that purifying selection was the main driver for the evolution of the *D. odorifera* R2R3-MYB gene (Table S2).

### 2.4. Evolutionary Relationship Analysis of D. odorifera and A. thaliana R2R3-MYB TFs

**Figure 4:**
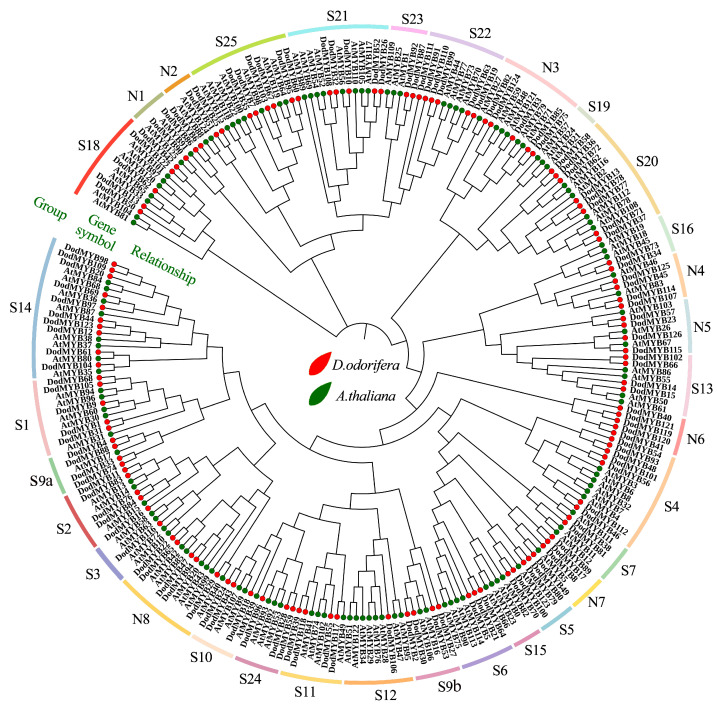
Phylogenetic tree of the R2R3-MYB transcription factor family of *D. odorifera* and *A. thaliana*. The *D. odorifera* MYB proteins were divided into 33 subfamilies (S1–25 and N1-8) based on the phylogenetic tree. The relevant biological functions for each subfamily are listed in Table S4. Red color indicates *D. odorifera*, green color indicates *A. thaliana*.

A NJ phylogenetic tree of R2R3-MYB genes from *D. odorifera* (126 members) and *A. thaliana* (126 members) was constructed to further investigate the evolution and functions of *D. odorifera* R2R3-MYB TFs (Figure 4 and Figure S4). The R2R3-MYB TFs of the same subfamily might have the same or similar functions. So far, the functions of the MYB TFs in *A. thaliana* are the most extensively mined, which can provide a reference basis for the functional delineation and naming of each subfamily of *D. odorifera* R2R3-MYB genes [20]. R2R3-MYB genes of *D. odorifera* were classified into 33 subfamilies with reference to their homology (Figure 4, Table S3). The name of subclade S1–S25 is based on the results of Dubos et al. for *A. thaliana* [8], and N1-N8 is an unnamed subclade in that study. Among these subfamilies, S19 comprised only *A. thaliana* R2R3-MYB members and N6 comprised only *D. odorifera* R2R3-MYB members (Figure 4), indicating the functional specificity among different plants. According to the classification results, among the S1–S25 subfamilies with more defined functions, nine subfamilies (S3–S7, S10, S12, S13, and S21) may be mainly involved in the metabolic processes of anthocyanins, flavonoids, and phenylpropanoids. The large amounts of flavonoids produced after heartwood formation may be associated with the genes of these subgroups. Another 16 subfamilies might be involved in processes such as defense responses (S1, S2, S11, S18, S20, S22, and S23) and development or tissue differentiation (S9, S14–16, S18, S19, S21, S24, and S25) in plants (Table S3). Protein sequences in the same subgroups were relatively conserved in structure and function, and these results suggested possible redundancy or overlap in the functions of *D. odorifera* R2R3-MYBs. The functions of *D. odorifera* S1–25 subfamily genes were predicted via a comparative phylogenetic analysis of *A. thaliana*, but the functions of subfamilies N1-8 could not be accurately predicted by examining *A. thaliana* alone because of the large number and complex types of R2R3-MYB families. The evolutionary analysis was performed using 150 R2R3-MYB transcription factor protein sequences of more than 40 plants based on some recent studies to further determine the functions of *D. odorifera* N1–N8 subfamily TFs (Figure S4). Among subfamilies N1-3, eight were associated with the growth, development, and stress response of plants, and subfamilies N4-7 were involved in the secondary metabolic processes of plants.

### 2.5. Expression Patterns of D. odorifera R2R3-MYB Genes in Different Tissues

**Figure 5:**
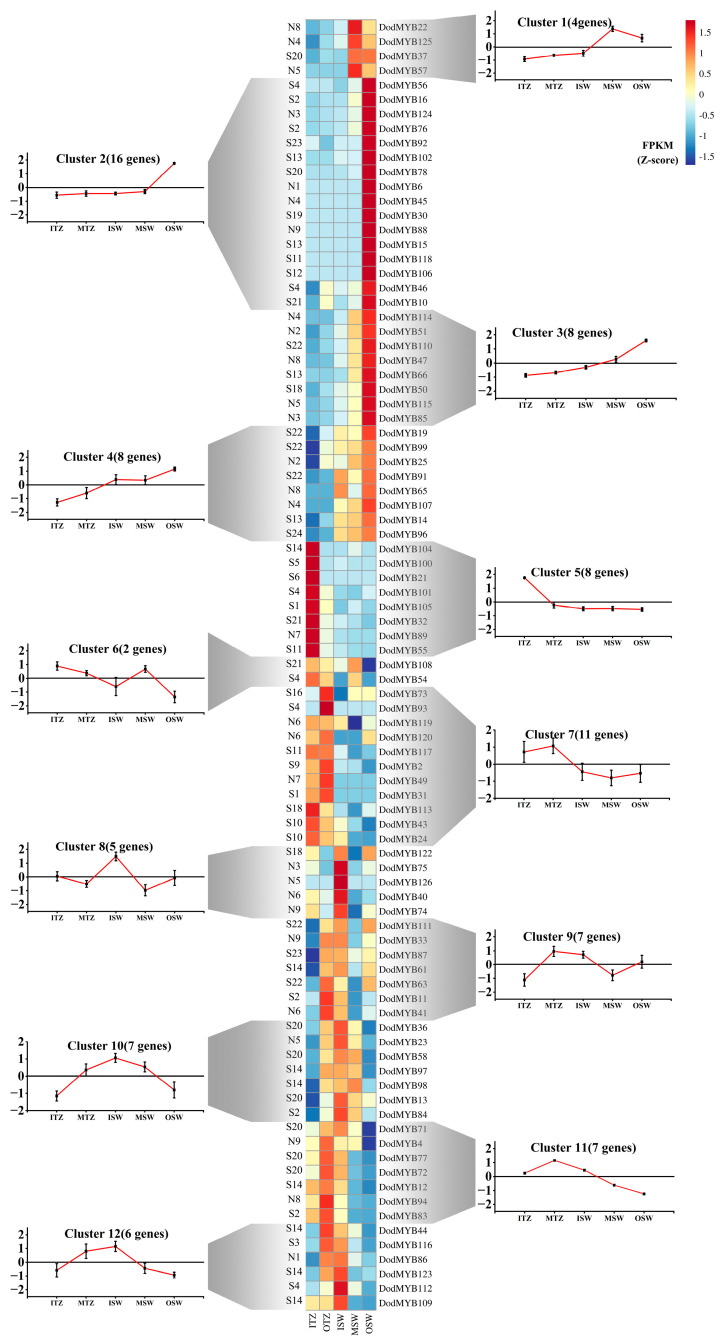
Expression patterns of MYB TFs in different parts of the xylem clustered in 12 expression groups. Transcript abundance is expressed in z-score standardized fragments per kilobase of exon per million fragments mapped (FPKM) values. For each gene, its name is shown to the right of the heatmap, whereas the short name of the phylogenetic subfamily is included to the left. Next to each RNAseq-based cluster, there is a graph with the mean transcript abundance ± SD for the entire cluster.

Based on published transcriptome expression abundance data of the tissues of roots, leaves, seeds, and stems in *D. odorifera* (Table S4) [3] and the supplemented data of different parts (outer sapwood, middle sapwood, inner sapwood, outer transition zone, and inner transition zone) of the xylem (Table S5), the expression patterns of R2R3-MYB in different tissues and organs were analyzed to explore the molecular functions so as to reveal the spatiotemporal expression pattern of *D. odorifera* R2R3-MYB TFs. Among the 126 DodMYB genes, most of the R2R3-MYB genes had a low abundance. Nine genes had undetectable expression in all tissues of *D. odorifera*. The remaining 117 family members were divided into five clusters according to their transcript abundance (Figure S5). The R2R3-MYB gene family showed tissue-specific expression, with most DodMYB genes preferentially expressed in the tissues of one part of the plant. For example, 46 genes (Cluster IV) were expressed at high levels in the roots, while 13 genes (Cluster I) and 17 genes (Cluster III) were preferentially expressed in the leaf and seed parts, respectively. Eighty-eight R2R3-MYB genes were expressed in different parts of the xylem, and there were significant differences in expression patterns (Figure 5). A total of 36 genes from Clusters 1–4 were preferentially transcribed in the outer and middle layers of the xylem sapwood, and a total of 14 genes from Clusters 9–10 were highly expressed in the inner sapwood and outer transition zones. Among these DodMYB TFs, eight genes (DodMYB21, DodMYB32, DodMYB55, DodMYB89, DodMYB100, DodMYB101, DodMYB104, and DodMYB105) were expressed in the xylem transition zone with high transcript abundance (FPKM > 100), presumably with possible relevance to xylem heartwood formation. The three genes DodMYB89, DodMYB32, and DodMYB55 (belonging to Cluster 12) had high transcript expression abundances (FPKM > 100) and were consistent with the results of the relative expression levels measured by RT-qPCR (Figure 5 and Figure S6).

### 2.6. Subcellular Location of DodMYB89

**Figure 6:**
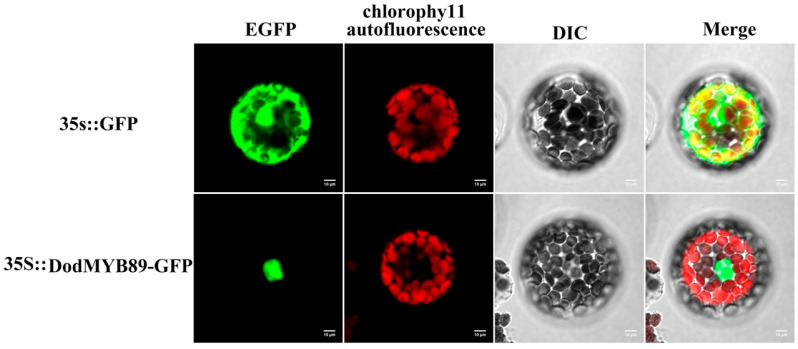
The subcellular localization of DodMYB89 in *A. thaliana* protoplast. EGFP: enhanced green fluorescent protein; 35::GFP: empty vector control group; 35S::DodMYB89-GFP:transient fluorescent expression of DodMYB89.

The results of the comparative phylogenetic analysis of the R2R3-MYB TFs (Section 2.4, DodMYB89 belongs to the N4 subfamily) and transcript expression abundance analysis of the R2R3-MYB genes in different parts of xylem (Section 2.5) suggest that DodMYB89 may be involved in regulating the biosynthesis of secondary metabolites during heartwood formation. To clarify the specific expression location of DodMYB89 in cells, the full-length protein-coding sequence of DodMYB89 was fused with the GFP sequence to construct a subcellular localization vector, and the recombinant plasmid was transformed into the protoplasts of *A. thaliana* leaves, and the transient expression phenomenon of DodMYB89 in cells was observed using laser confocal microscopy. The results showed that the green fluorescence of the GFP empty vector was observed throughout the cells with no obvious subcellular localization specificity, whereas a green fluorescent signal was observed in the protoplasts for the DodMYB89-containing fusion protein, indicating that the protein encoded by the DodMYB89 gene was mainly localized in the nucleus (Figure 6).

### 2.7. Validation of Transcriptional Activation Activity of DodMYB89

**Figure 7:**
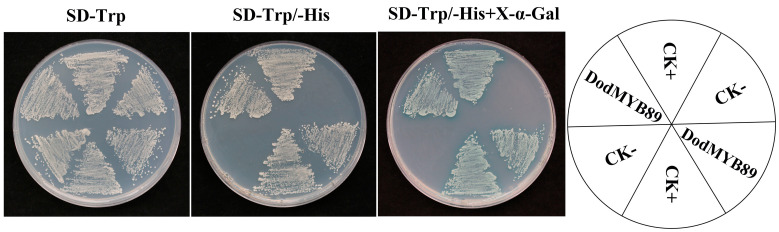
Validation of the transcriptional activation activity of DodMYB89. SD-Trp/His is a defective medium, lacking tryptophan (Trp) or histidine (His). X-α-Gal: 5-Bromo-4-chloro-3-indoxyl-α-D-galactopyranoside. CK+ means positive control check and CK− means negative control check.

To validate the transcriptional activity of DodMYB89, the yeast GAL4 system was used. pGBKT7-VP16 was used as a positive control check. On SD-Trp-deficient medium, the spots of yeast could grow normally, indicating that the plasmid was successfully cotransformed into yeast cells; on SD-Trp/-His, the transcription factor had transcriptional activation activity and was able to activate the expression of downstream reporter genes, so the spots could grow. On SD-Trp/-His + X-α-Gal plates, the transcription factor could activate the reporter gene and cause β-galactosidase catabolism, reacting with X-α-gal to show a blue color (Figure 7). pGBKT7 was a negative control check. The spots of yeast could grow normally on SD-Trp-deficient medium, indicating successful co-transfection of the plasmid into yeast cells; spots could not grow on SD-Trp/-His and SD-Trp/-His + X-α-Gal as there was no transcriptional activation activity to activate the transcription of the downstream reporter gene. It was also unable to interact with X-α-Gal. The experimental group pGBKT7-DodMYB89 was able to grow on SD-Trp/-His and turn blue on SD-Trp/-His + X-α-Gal plates, demonstrating the transcriptional activation activity of the transcription factor DodMYB89.

### 2.8. DodMYB89 Affects the Biosynthesis of Flavonoid Components

**Figure 8:**
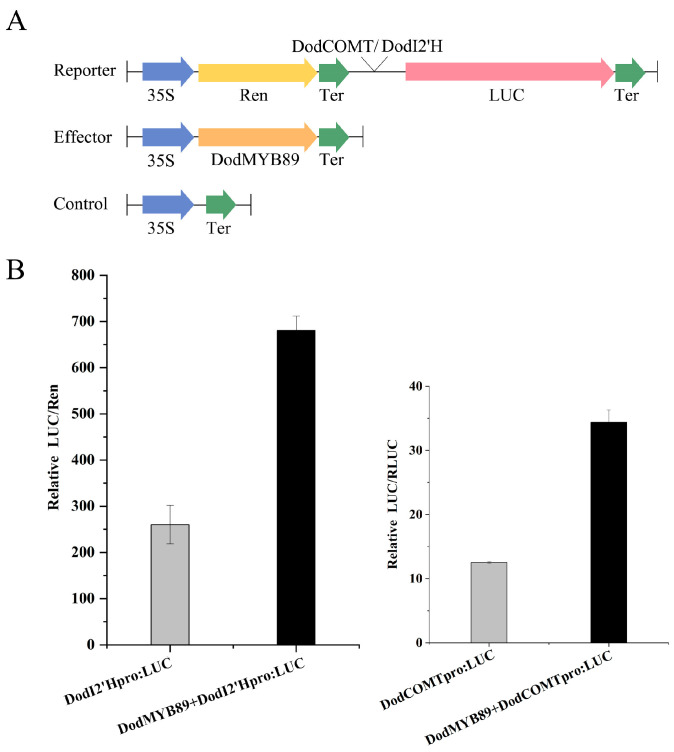
Activation assays of *DodI2′H* and *DodCOMT* promoters by *DodMYB89* transcription factors using LUC reporter. (**A**) Schematic diagram of the effector and reporter plasmids used in the transient assay in leaf epidermal cells of *Nicotiana benthamiana*. LUC, firefly luciferase. Ren, Renilla luciferase; (**B**) *DodMYB89* activates the transcription of *DodCOMT* and *DodI2’H*. LUC activity was normalized to REN activity as an internal control.

The transcript expression of *DodI2’H* (isoflavone 2’-hydroxylase gene) and *DodCOMT* (caffeic acid O-methyltransferase gene) was upregulated 88-fold and 26-fold, respectively, during heartwood formation (ITZ vs. OSW, Table S6), both of which are candidate structural genes of flavonoid synthesis. In order to investigate the regulatory role of the transcription factor DodMYB89 on *DodCOMT* and *DodI2’H*, the promoter sequences of the two structural genes were cloned with reference to the genome sequence of *D. odorifera*, and the cis-acting elements present in the promoter sequences were analyzed. The promoter sequences of both genes were found to contain MYB binding sites (Figure S7), so it is hypothesized that DodMYB89 may have some transcriptional activation effect on the promoter regions of *DodI2’H* and *DodCOMT*. A dual-luciferase reporter assay was performed in leaves of tobacco by cloning the promoter sequences of the two structural genes with reference to the *D. odorifera* genome sequence (Figure 8A). The *DodCOMT* and *DodI2‘H* promoters were used as positive controls, respectively. The results showed that injection of the transcription factor DodMYB89 significantly increased the activity of the *DodCOMT* and *DodI2’H* promoters by 2.74- and 2.61-fold, respectively (Figure 8B).

## 3. Discussion

The publication of *D. odorifera* genomic data helped this study to identify and functionally mine the *D. odorifera* R2R3-MYB transcription factor family for the first time [3]. Most plant species had between 50 to 200 R2R3-MYB TFs [7]. For example, *A. thaliana* comprised 126 members [8], *Casuarina equisetifolia,* 107 members [5]; *Eucalyptus grandis,* 141 members [9]; and *Populus trichocarpa,* 196 members [10]. However, the number, type, and function (especially related to heartwood formation) of 2R-MYB in *D. odorifera* are currently unknown.

This study screened 126 R2R3-MYB genes in the *D. odorifera* genome. R2R3-MYB proteins had different physicochemical properties (Table S1), which implied the functional diversity of R2R3-MYB family members. Chromosome location analysis showed that the DodMYB gene was distributed non-homogeneously on chromosomes in clusters, which might be due to the nonhomogeneous replication events of chromosome segments (Figure S1). Gene duplication events were the main mechanisms that promoted the evolutionary expansion and diversification of the R2R3-MYB gene family [21]. The analysis of the collinearity results showed that the six R2R3-MYB genes had three tandem repeats on two chromosomes (chromosomes 2 and 10). Moreover, 55 pairs of segmental duplication involving 87 genes were identified on 10 chromosomes (Figure 3; Table S2), indicating that this event might play a key role in amplifying *D. odorifera* R2R3-MYB genes. R2R3-MYB TFs with collinearity relationships show similar gene structures and motif compositions, and these genes often belong to the same branch. For example, DodMYB55 and DodMYB117, with collinearity relationships to the same S11 subclade, have the same motif number and type and contain three exons and two introns, which is the most common intron–exon structural pattern (Figure 3). Hence, gene duplication expanded the R2R3-MYB family, giving rise to diversity in the gene structure and functional divergence that may contribute to plant adaptation to the environment. However, the similarity in structure shared by homologous subfamily genes can provide a basis for constructing phylogenetic relationships and exploring R2R3-MYB gene functions.

The protein sequences of R2R3-MYBs from *D. odorifera* and *A. thaliana* were co-constructed into a phylogenetic tree to investigate the potential functions of *D. odorifera* R2R3-MYB TFs (Figure 4). Genes with similar biological functions tended to cluster in the same subfamily, and the regularity of gene expression patterns in different tissues in relation to phylogeny was combined to find out the functions exercised by R2R3-MYB genes in plant growth and development. The functions of approximately 80% of R2R3-MYB TFs in the model plant *A. thaliana* have been characterized [7]. The *D. odorifera* R2R3-MYB gene family was divided into 33 subfamilies with reference to the classification of *A. thaliana* R2R3-MYB family members, and the specific physiological functions of DodMYBs were examined (Table S1). For example, DodMYB35, DodMYB60, and DodMYB7 of the S25 subfamily show high transcript expression levels at the seed site and share homology with AtMYB115 and AtMYB118, which can regulate the formation of the embryo in *A. thaliana*, implying that these three genes are likely to be involved in the developmental regulation of seeds [22,23]. Similarly, Cluster Ⅳ comprised 46 genes specifically expressed in roots. Among the 31 genes, DodMYB12, DodMYB20, DodMYB44, DodMYB69, DodMYB98, DodMYB109, and DodMYB123 belonged to the S14 subfamily, a subfamily of genes associated with root elongation and development [24,25].

As a perennial woody plant, the main economic value of *D. odorifera* lies in the xylem, especially in the heartwood part of the xylem. The scientific regulation of heartwood formation becomes the key to increasing the forest production value of *D. odorifera* with the increase in its plantation area. The heartwood is formed after the programmed death of parenchyma cells in the transition zone and the aggregation of secondary metabolic material in the heartwood. Five regions of the xylem, outer sapwood layer, inner sapwood layer, outer transitional zone, middle transitional zone, and inner transitional zone (near the heartwood), were selected for transcriptome sequencing to obtain the relative expression of the MYB gene and perform cluster analysis to explore the molecular regulation mechanism of xylem development to programmed death. A total of 36 genes in Clusters 1–4 were specifically expressed in the outer layer of the xylem sapwood. The mature sapwood had basically completed the developmental processes such as cell elongation and cell wall thickening, and its active cells mainly maintained their own primary metabolism (DodMYB18 belonged to the subfamily S10) and their own defense against stress (DodMYB50 belonged to the subfamily S18, DodMYB16 and DodMYB76 belonged to S2, and DodMYB37 and DodMYB78 belonged to subfamily S20), where S2, S10, S18, and S20 were associated with plant metabolism and defense against endogenous stresses [16,26,27]. The changes in cellular physiological activity occurring in parenchyma cells in the transition zone were closely related to heartwood formation.

The genes associated with Clusters 9–10 were highly expressed in the outer transition zone and in the inner sapwood. The most numerous genes were associated with cellular defense in this cluster, with three genes of the S20 subfamily (DodMYB36, DodMYB58, and DodMYB13), two genes of the S2 subfamily (DodMYB11 and DodMYB84), and two genes of the S22 subfamily (DodMYB63 and DodMYB111). The low number of metabolism-related genes in this cluster suggested that this location was not the site of secondary metabolite production, although the physiological activity of the cells was reduced. The transition zone layer was the region closest to the heartwood, and Cluster 11 (six genes) was specifically expressed in this region. However, the expression of DodMYB100, DodMYB101, DodMYB104, DodMYB105, and DodMYB21 was low (FPKM < 5), presumably with little correlation with the physiological changes in this region. In contrast, the expression of the remaining three genes at this site was extremely high (FPKM > 100). The DodMYB32 transcription factor (FPKM > 1000) belonged to the S21 subfamily and the genes of this subfamily (AtMYB52, AtMYB54, and AtMYB69) were associated with the thickening of the cell wall [27], which was consistent with previous studies on the upregulated expression of genes in the transition zone during heartwood formation associated with the deposition of secondary walls [28]. In recent years, this subclade of genes has been found to regulate the production of terpene metabolic pathways. It is hypothesized that the production of a large number of aroma components such as nerolidols, which are rich in heartwood, may also be regulated by this gene. DodMYB89 was specifically expressed in the inner transition zone with a very high expression level (FPKM > 1000) and was closely related to the AtMYB5 (N7b) branch of *A. thaliana*. This subclade of genes regulated the secondary metabolism, such as phenol propane metabolism, flavonoid, and anthocyanin syntheses. DodMYB89 also has a high structural similarity to the conserved domain of the Gm03g258700 (N7a) protein (Figure S4), a homologue of a gene with the function of regulating isoflavone synthesis [29]. This result suggests that the secondary metabolites in *D. odorifera* heartwood may be synthesized in situ within the transition zone layer, rather than in sapwood for transport to the inner xylem layer. MYB TFs (DodMYB32, DodMYB55, and DodMYB89) might play important roles in *D. odorifera* heartwood formation. These results might help mine the molecular function of DodMYBs to identify and find TFs associated with heartwood formation.

Further experimental studies were conducted to verify the role of the DodMYB89 transcription factor in the formation of *D. odorifera* heartwood. DodMYB89 is localized in the nucleus and is a transcriptional activator that positively regulates the activity of the promoters of structural genes (*DodI2’H* and *DodCOMT*) in the flavonoid synthesis pathway. All these results suggest that DodMYB89 is involved in regulating the production of secondary metabolites in heartwood.

## 4. Materials and Methods

### 4.1. Screening of the R2R3-MYB Transcription Factor Family in the D. odorifera Genome

The hidden Markov model of the MYB protein structural domain (PF00249) was used as a search file to initially screen candidate MYB genes (criteria E < 10^−5^) from the *D. odorifera* genomic data (http://gigadb.org/dataset/100760, accessed on 3 October 2022) using HMMER 3.0 software. Then, we used the CD-HIT online suit (http://weizhong-cluster.ucsd.edu/cdhit-web-server/cgi-bin/index.cgi?cmd=cd-hit, accessed on 4 October 2022) to remove redundant sequences [30]. Finally, the candidate MYB sequences were further manually examined for the completeness of the conserved domains in the protein sequences using the Pfam database (https://pfam.xfam.org/, accessed on 4 October 2022) and the NCBI Conserved Domain database (https://www.ncbi.nlm.nih.gov/Structure/bwrpsb/bwrpsb.cgi, accessed on 4 October 2022) [31]. The structural domain position information files were downloaded for gene structure analysis. The R2R3-MYB genes were named DodMYB1–DodMYB126 according to their order of location on the chromosomes of *D. odorifera*. The physicochemical properties such as the molecular mass, isoelectric point, and instability coefficient of *D. odorifera* MYB proteins were analyzed online the Expasy database (https://web.expasy.org/, accessed on 6 October 2022), and subcellular localization prediction analysis was performed using the WOLF PSORT online website (https://wolfpsort.hgc.jp/, accessed on 6 October 2022).

### 4.2. Conserved Motif of Protein and Gene Structure Analysis of R2R3-MYB TFs

To further investigate the distribution, alignment, and conserved characteristics of the MYB protein sequence, the conserved motifs were analyzed by MEME Suite 5.4 online website (https://meme-suite.org/meme/tools/meme, accessed on 6 October 2022) [32]. The number of conserved motifs searched was 10, and the rest of the settings were left unchanged. The exon/intron gene structure and conserved motif features were visualized simultaneously using the built-in function (Gene Structure View) of TBtools software v1.0987663 [33].

### 4.3. Chromosome Localization and Covariance Analysis

The location of each MYB gene on the chromosome was obtained from the *D. odorifera* genome using TBtools software v1.0987663 [33], and the data were mapped to the chromosome using the built-in tool (Gene Location Visualize). The duplication events and synteny relationships of the R2R3-MYB gene members were identified by using One Step MCscanX program of TBtools software v1.0987663 with the default parameters and finally visualized and analyzed on a Circos tool [34].

### 4.4. Phylogenetic Analysis

The phylogenetic evolutionary trees were constructed by combining the MYB protein sequences from *A. thaliana* or other plants with *D. odorifera* using the Molecular Evolutionary Genetics Analysis Version 7.0 (MEGA) software [35]. Firstly, ClustalW tool was used for multiple-sequence alignment, and then the neighbor-joining method (NJ) was used to construct the evolutionary tree with the following parameters: Poisson correction, pairwise deletion (50%), and bootstrap analysis with 1000 replicates. Finally, the evolutionary tree was embellished using the Interaction Tree of Life online website (https://itol.embl.de/, accessed on 6 October 2022) [36].

### 4.5. Plant Material and Sample Preparation

Three *D. odorifera* trees with the same growth potential for 12 years were selected and collected from the nursery of the Guangxi University, Nanning, Guangxi Zhuang Autonomous Region in June 2021. The trees were cut and sawed into disks using a chainsaw, and the disks were quickly trimmed into long strips using a chisel. The extent of the block in the radial direction included the five parts: outer sapwood (OSW), middle sapwood (MSW), inner sapwood (ISW), outer transition zone (OTZ), and inner transition zone (ITZ) (Figure S8). The blocks were then frozen using liquid nitrogen and transported on dry ice to the laboratory for storage in a refrigerator at −80 °C.

### 4.6. RNA Extraction and Transcriptome Sequencing

Samples from different parts of the xylem were confirmed, and then RNA was extracted by Gene Deveo Co., Ltd. (Guangzhou, China). Each part was used as a sample, and three biological replicates of the experiment were performed for each sample. The procedure is briefly described below. The frozen samples were taken out of the ultra-low temperature refrigerator. The samples were then quickly ground into a powder using liquid nitrogen and 1 mL of the extraction buffer (4M isothiocyanate, 25 mM sodium citrate, 5% SDS, 20% PVP, and 1% β-ME) was added. The powder was then vortexed and mixed well, and then 100 µL of 2M NaAc and 1 mL of chloroform:isoamyl alcohol (24:1) were added to it. The mixture was centrifuged at 4 °C for 15 min at a speed of 13,400× *g*. After centrifugation, the supernatant was transferred to a new RNAase-free centrifuge tube. Isopropanol was added to it in the same proportion, and the mixture was kept at −20 °C for 3–4 h. After centrifugation, the supernatant was aspirated. Chilled 70% ethanol was added to the aspirated supernatant, washed two to three times, and dried at room temperature. An appropriate volume of diethyl pyrocarbonate was then added to it. The transcriptome sequencing and library construction were performed by the Gene Deveo Co., Ltd. Furthermore, three key genes (DodMYB89, DodMYB32, and DodMYB55) were selected and analyzed by quantitative real-time RT-PCR (qRT-PCR). The sequence information of the specific primers was designed using Primer Premier software 5.0 and listed in Table S7. The relative gene expression level was calculated by the 2^−ΔΔCt^ method and normalized to the housekeeping gene *HIS2* with three technical replications [37].

### 4.7. Subcellular Localization

The subcellular localization of DodMYB89 was identified using a transient assay based on the *A. thaliana* protoplast. The CDSs of DodMYB89 genes were amplified and cloned into vector pAN580 by a homologous recombination reaction. The recombinant vector was transformed into *Agrobacterium* tumefaciens strain GV3101. The primers used are listed in Supplementary Table S1. *Agrobacterium* transformants were infiltrated into the protoplasts of *A. thaliana,* and the fluorescence of GFP was observed after 18–24 h of incubation as previously described [38].

### 4.8. Transcriptional Activation Activity of DodMYB89

The coding sequences of *DodMYB89* was cloned into the pGBKT7 vector to obtain the recombinant protein. The pGBKT7-VP16 (positive control), pGBKT7 (negative control), and pGBKT7-419.222 plasmids were transformed into *Saccharomyces cerevisiae* AH109 competent cells and coated on SD-Trp plates, and when the yeast spots grew to 2–3 mm in size, the spots were picked and mixed in 100 µL of 0.9% NaCl solution. The three bacterial solutions were coated on SD-Trp, SD-Trp/-His, and SD-Trp/-His+X-α-Gal solid media, respectively, and incubated in an incubator at 30 °C for 3–5 d (inverted) to observe the growth of yeast.

### 4.9. Transient Dual-Luciferase Assays

The cis-acting elements of *DodI2′H* and *DodCOMT* were predicted in the 2000-bp upstream regions with the plantCARE website (http://bioinformatics.psb.ugent.be/webtools/plantcare/html/, accessed on 20 October 2022). Transient dual-luciferase assays were performed as previously described [39]. The promotors of *DodI2′H* and *DodCOMT* were cloned into pGreenII-0800-luc vectors to construct reporter vectors. The coding sequences of *DodMYB89* was cloned into the GreenII-62-SK vector to obtain the recombinant protein. All the recombinant vectors were transformed into *Agrobacterium Tumefaciens* GV3101-pSoup competent cells. The resuspensions were mixed, and the 62-SK empty resuspensions were mixed with the promoter resuspensions as controls. *Nicotiana benthamiana* leaves were injected and incubated in a smart artificial climate chamber for 3 d. The leaves were collected and analyzed for luciferase activity using the TransDetect^®^ Double-Luciferase Reporter Assay Kit (TransGen Biotech Co., LTD, Beijing, China) according to the manufacturer’s instructions, and the experiment was repeated three times.

## 5. Conclusions

The R2R3-MYB transcription factor family was comprehensively analyzed based on the *D. odorifera* genome. A total of 126 DodR2R3-MYB TFs were identified and classified into 33 subfamilies based on phylogenetic relationships, with transcription factor proteins related to the flavonoid biosynthesis pathway mainly concentrated in the subfamilies S3-7, 10, 12-13, 21, and N4-7. *D. odorifera* MYB TFs were clustered on 10 chromosomes. Fifty-eight collinearity MYB gene pairs were identified in the *D. odorifera* genome, and fragment duplication events significantly expanded the *D. odorifera* MYB gene family. Three R2R3-MYB TFs (DodMYB89, DodMYB32, and DodMYB55) specifically transcribed and expressed in the ITZ of the xylem were associated with heartwood formation. Among them, the DodMYB89 TF, localized in the nucleus, has transcriptional activation activity and can regulate the activity of the *DodCOMT* and *DodI2′H* promoters, thereby affecting the synthesis of flavonoids during heartwood formation.

## Data Availability

The original contributions presented in the study are included in the article/supplementary materials. Further inquiries can be directed to the corresponding authors.

## Supplementary Materials

The supporting information can be downloaded at: https://www.mdpi.com/article/10.3390/ijms241512430/s1.
