# Supplementary material for: Function of the R2R3-MYB Transcription Factors in Dalbergia odorifera and Their Relationship with Heartwood Formation
Source: Int J Mol Sci. 2023 Aug 4;24(15):12430. doi: 10.3390/ijms241512430 (PMC10419101; doi:10.3390/ijms241512430)
Supplement: Supplementary file 1 [file ijms-24-12430-s001.zip › Supplemental Figure S1-8.pdf]

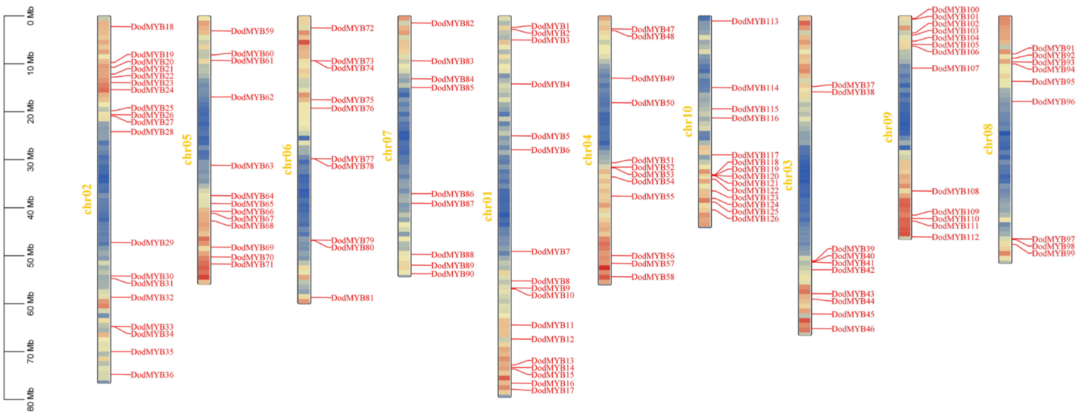

Figure S1. Location of genes on chromosomes

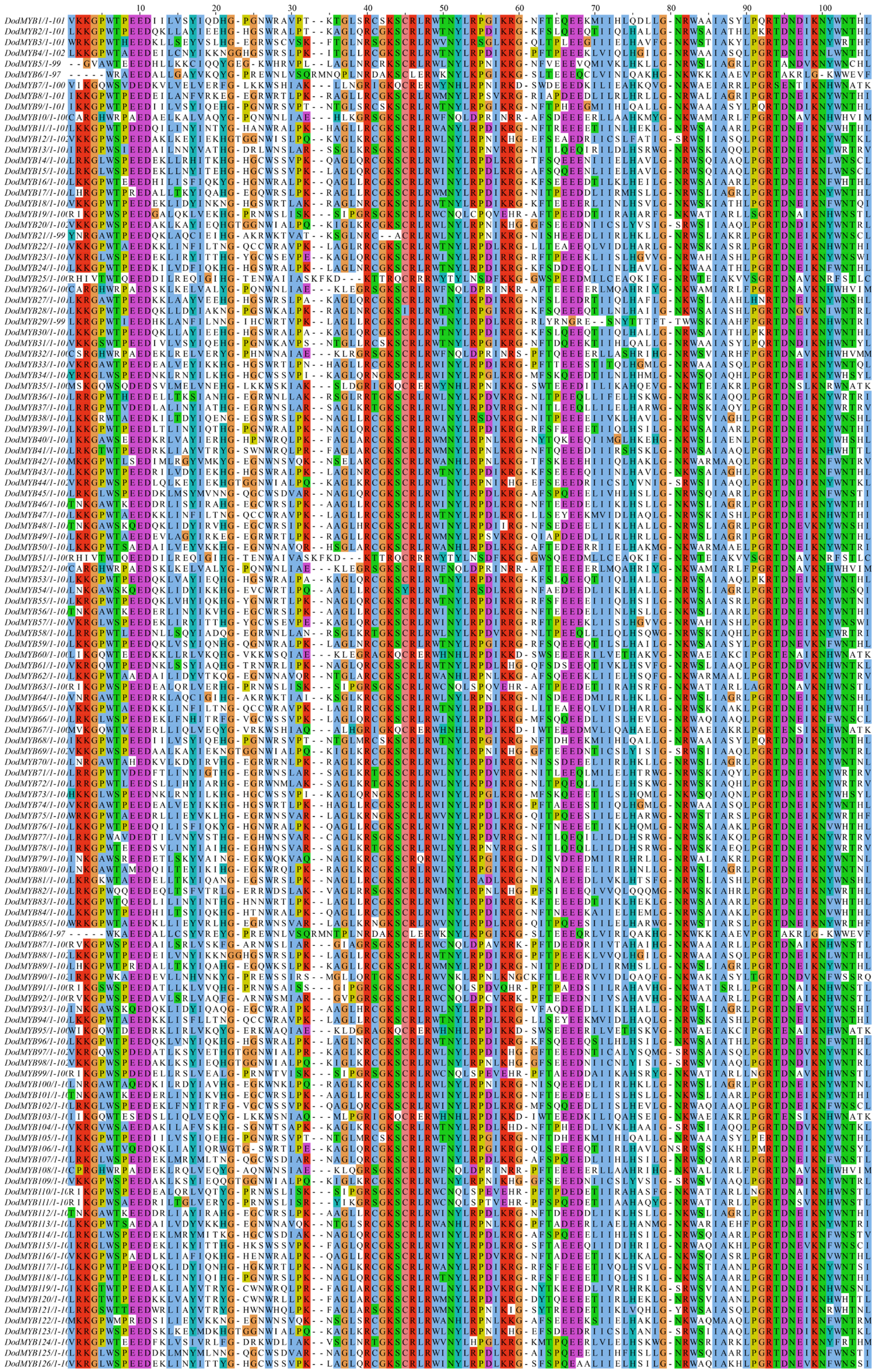

Figure S2. Results of multiple sequence alignment of 126 DodMYB TF.

	logo	E-value	Sites	Width
1.	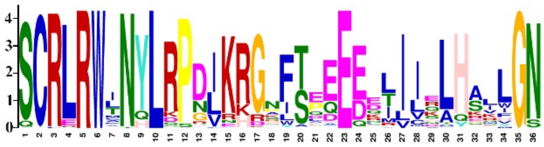	1.1e-3152	124	36
2.	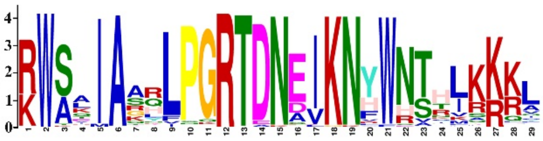	1.7e-2760	126	29
3.	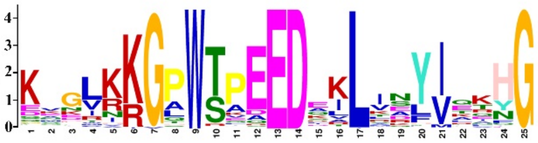	2.2e-1614	122	25
4.	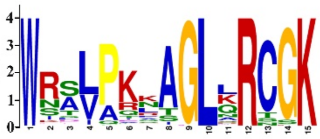	6.9e-947	101	15
5.	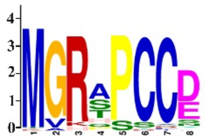	7.6e-308	65	8
6.	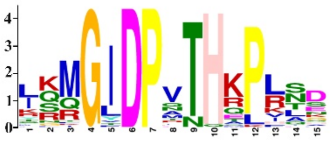	4.8e-218	38	15
7.	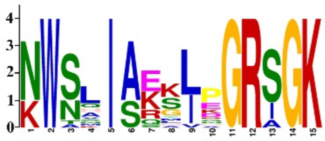	2.3e-102	19	15
8.	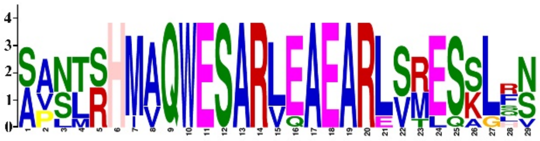	3.0e-091	8	29
9.	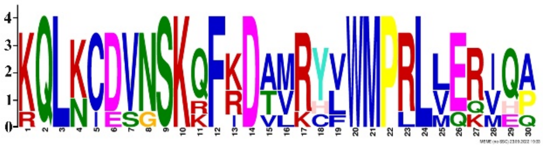	1.5e-068	6	30
10.	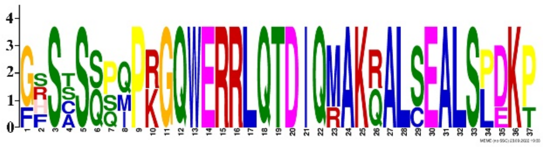	5.5e-040	4	37

Figure S3. Consensus sequences of motifs in R2R3-MYBs.

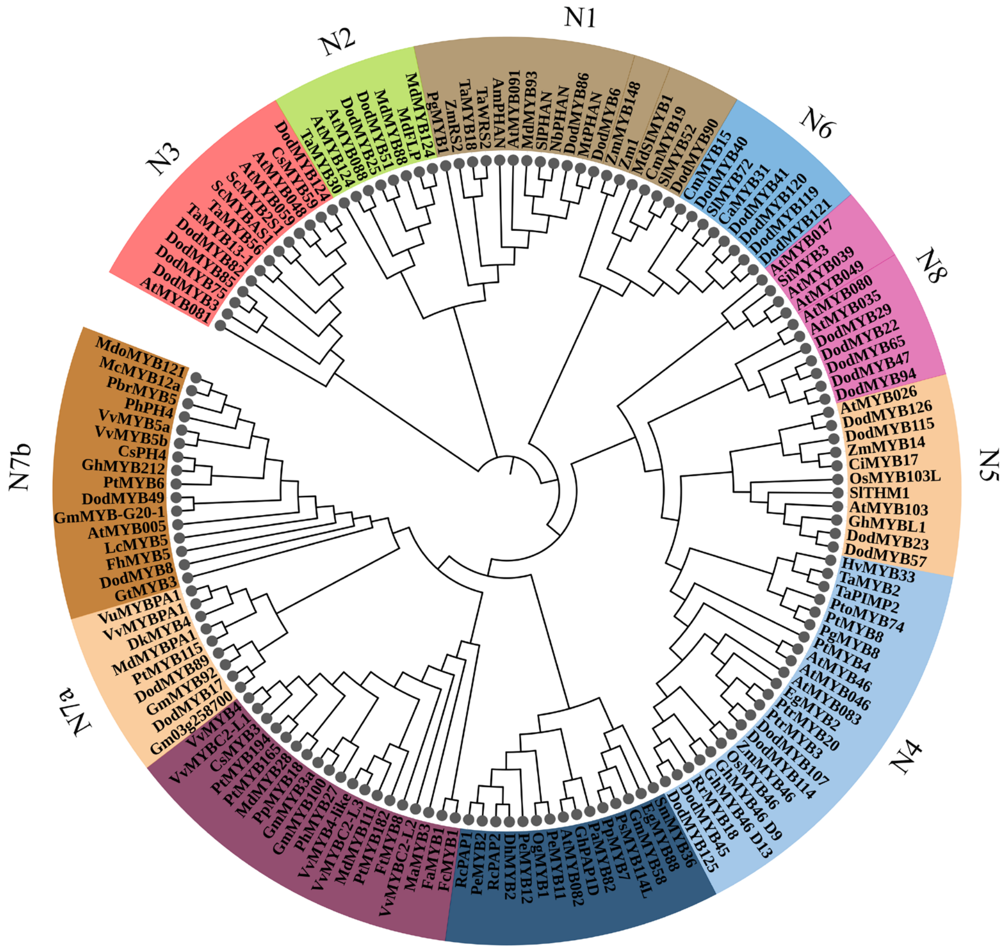

Figure S4. Developmental evolutionary tree analysis of the N1-8 subclade R2R3-MYB transcription factors. The amino acid sequence was derived from *Antirrhinum majus*, *Arabidopsis thaliana*, *Antirrhinum majus*, *Capsicum annuum*, *Cattleya hybrid*, *Chrysanthemum morifolium*, *Chrysanthemum morifolium*, *Cichorium intybus*, *Citrus sinensis*, *Citrus sinensis*, *Dendrobium hybrids*, *Diospyros kaki*, *Eucalyptus grandis*, *Eucalyptus gunnii*, *Fragaria ananassa*, *Fragaria chiloensis*, *Freesia hybrida*, *Gentian tutea*, *Glycine max*, *Gossypium hirsutum*, *Hordeum vulgare*, *Litchi chinensis*, *Malus domestica*, *Malus hybrid cultivar*, *Marchantia polymorpha*, *Medicago truncatula*, *Musa acuminata*, *Nicotiana benthamiana*, *Oncidium spp.*, *Oryza sativa*, *Paeonia suffruticosa*, *Panax ginseng*, *Petunia hybrida*, *Phalaenopsis equestris*, *Pinus pinaster*, *Pinus taeda*, *Platanu acerifolia*, *Populus tomentosa*, *Prunus persica*, *Pyrus betulaefolia*, *Rosa rugosa*, *Saccharum officinarum*, *Salvia miltiorrhiza*, *Selaginella moellendorffii*, *Setaria italica*, *Solanum lycopersicum*, *Tartary buckwheat*, *Trifolium arvense*, *Vaccinium uliginosum*, *Vitis vinifera*, *Zea mays*.

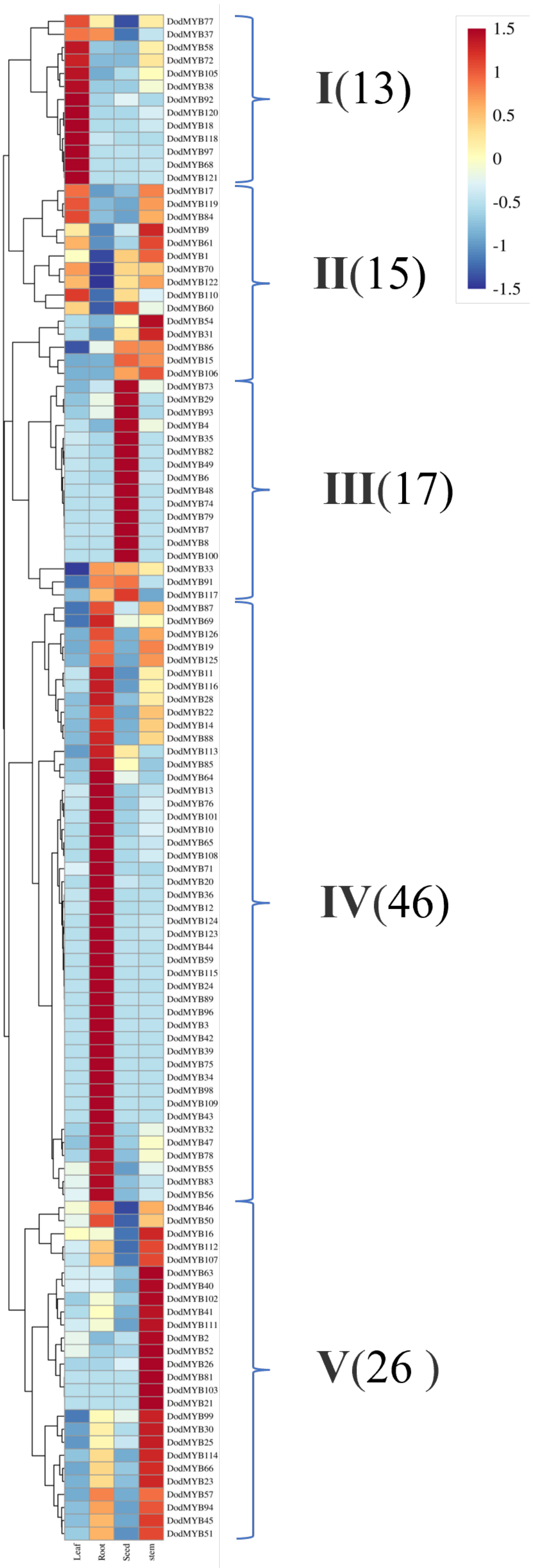

Figure S5. Expression patterns of MYB TFs in different parts of the xylem clustered in 12 expression groups. Transcript abundance is expressed in z-score standardized reads per kilobase of exon per million reads mapped (FPKM) values.

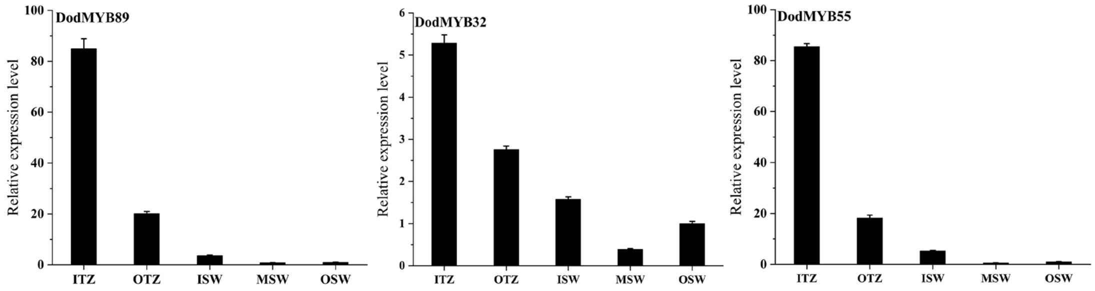

Figure S6. The expression pattern of DodMYB89, DodMYB32 and DodMYB55 in different parts of xylem. All data are expressed as the means ± SD (3 technical repetitions).

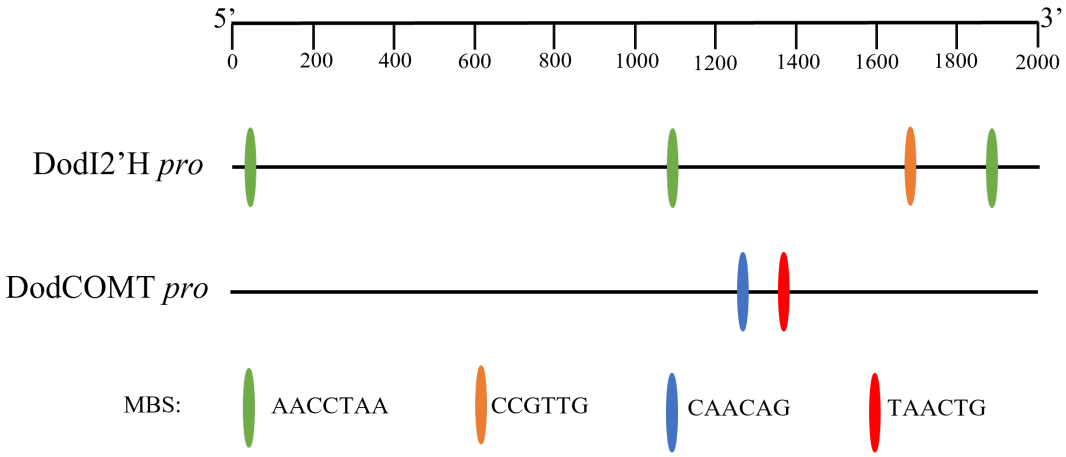

Figure S7. The schematic drawing of promoters and analysis the cis-regulatory elements for binding of MYB proteins. MBS: MYB binding site.

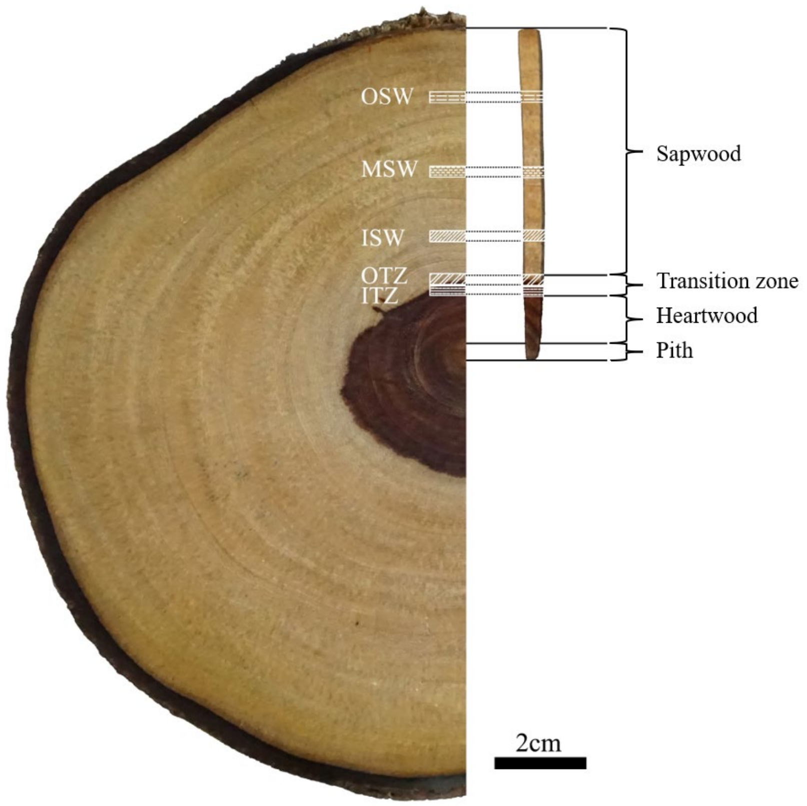

Figure S8. Cross section of a stem from *D. odorifera* tree showing the different wood tissues: outer sapwood (OSW), middle sapwood (MSW), inner sapwood (ISW), outer transition zone(OTZ), inner transition zone (ITZ).
